# Practical Qualitative Evaluation and Screening of Potential Biomarkers for Different Parts of *Wolfiporia cocos* Using Machine Learning and Network Pharmacology

**DOI:** 10.3389/fmicb.2022.931967

**Published:** 2022-07-08

**Authors:** Lian Li, ZhiTian Zuo, YuanZhong Wang

**Affiliations:** ^1^Medicinal Plants Research Institute, Yunnan Academy of Agricultural Sciences, Kunming, China; ^2^College of Traditional Chinese Medicine, Yunnan University of Chinese Medicine, Kunming, China

**Keywords:** *Macrohyporia cocos*, machine learning, fingerprint, network pharmacology, potential biomarkers

## Abstract

*Wolfiporia cocos* is a widely used traditional Chinese medicine and dietary supplement. Artificial intelligence algorithms use different types of data based on the different strategies to complete multiple tasks such as search and discrimination, which has become a trend to be suitable for solving massive data analysis problems faced in network pharmacology research. In this study, we attempted to screen the potential biomarkers in different parts of *W. cocos* from the perspective of measurability and effectiveness based on fingerprint, machine learning, and network pharmacology. Based on the conclusions drawn from the results, we noted the following: (1) exploratory analysis results showed that differences between different parts were greater than those between different regions, and the partial least squares discriminant analysis and residual network models were excellent to identify *Poria* and *Poriae* cutis based on Fourier transform near-infrared spectroscopy spectra; (2) from the perspective of effectiveness, the results of network pharmacology showed that 11 components such as dehydropachymic acid and 16α-hydroxydehydrotrametenolic acid, and so on had high connectivity in the “component-target-pathway” network and were the main active components. (3) From a measurability perspective, through orthogonal partial least squares discriminant analysis and the variable importance projection > 1, it was confirmed that three components, namely, dehydrotrametenolic acid, poricoic acid A, and pachymic acid, were the main potential biomarkers based on high-performance liquid chromatography. (4) The content of the three components in *Poria* was significantly higher than that in *Poriae* cutis. (5) The integrated analysis showed that dehydrotrametenolic acid, poricoic acid A, and pachymic acid were the potential biomarkers for *Poria* and *Poriae* cutis. Overall, this approach provided a novel strategy to explore potential biomarkers with an explanation for the clinical application and reasonable development and utilization in *Poria* and *Poriae* cutis.

## Introduction

*Wolfiporia cocos* (F. A. Wolf) Ryvarden & Gilb. is a medicine and food homologous of fungi belonging to the family Polyporaceae, named as “Indian bread” in North America and “Hoelen” in Japan (Jia and Zhang, 2016; Dong et al., 2017; Li et al., 2022b). Multiple pharmacological effects of *W. cocos* possess antitumor, anti-oxidation, anti-inflammatory, antibacterial activity, immunomodulatory, and immunity enhancements (Dong, 2015; Chen et al., 2019). *W. cocos*, as one of the edible and medicinal mushrooms, is widely recognized as a natural resource with proven health benefits and has traditionally played an important role in food, pharmaceuticals, and agriculture worldwide, which also used as one of the most common materials in traditional medicine in China and some other Asian countries. In fact, wild coconut palms are rare in nature and therefore have long been prized and cultivated varieties in commercial demand. *W. cocos* are often divided into two types according to different parts and functions: *Poriae* cutis and *Poria*. The *Poriae* cutis is the outer peel part of *W. cocos*, which is beneficial due to its diuretic effect, whereas the white part of *W. cocos* named *Poria* has a long history in food and medicine due to its effect in invigorating and tonifying the spleen, soothing the spirit, and tranquilizing the heart (Commission, 2020). The diuretic effect is gradually weakened, and the effect of tranquilizing the mind and the nerves is enhanced. A study proved that the diuretic effect was enhanced whereas the sedative effect was weakened from the inside to the outside of *W. cocos* Qian et al., 2018). In recent years, research on various aspects of *W. cocos* have gradually increased, and its medicinal effects and edible aspects have attracted much attention, but the differences in the chemical components of different medicinal parts are still unclear. In addition, “*Chinese Pharmacopeia*” of the 2020 edition does not record “content determination” for both *Poria* and *Poria*e cutis (Commission, 2020), and there is a lack of practical evaluation methods for the rational use of *Poria* and *Poriae* cutis. Therefore, it is necessary to screen potential biomarkers of *Poria* and *Poriae* cutis of *W. cocos* for distinguishing parts and quality evaluation.

*W. cocos* is rich in a variety of chemical components, among which triterpenes and polysaccharides are the main active components. Triterpenes are important secondary metabolites and are also the material basis for the efficacy of *W. cocos*. The components of dehydrotumulosic acid, poricoic acid A, dehydropachymic acid, pachymic acid, and dehydrotrametenolic acid are the main potential biomarkers of *W. cocos* (Qian et al., 2018.). They are not considered as the indicator components here due to the natural polysaccharide components being generally insoluble in water and having low biological activity. The formation of secondary metabolites of medicinal plants is closely related to their individual development process. A report showed that the content of triterpene medicinal parts of *W. cocos* was significantly different to be determined and analyzed by UPLC-UV-MS (Li, 2013). Alatengqimuge et al. (2008) determined the concentration of triterpenes in rat plasma by the high-performance liquid chromatography-ultraviolet detection method, which provided a scientific basis for the pharmacodynamics of *W. cocos*. Although the above method can determine the triterpenes in different parts of *W. cocos*, these methods are expensive, time-consuming, and complicated to operate. Therefore, the triterpenoids of *W. cocos* should be used as the reference for the selection of potential biomarkers with the technology of superior characteristics such as easy to operate, fast, and inexpensive.

As an emerging field of pharmacology, network pharmacology emphasizes the concept of “network target, multi-component therapy” and the holistic thinking shared by traditional Chinese medicine (TCM) (Zhang et al., 2013). A summary of typical application scenarios of network pharmacology analysis is as follows: (1) drug-target prediction; (2) analysis of materials and the action mechanism of TCM syndromes and prescriptions; (3) analysis of TCM quality markers; and (4) study on the compatibility laws of TCM prescriptions, among others. Network pharmacology affords new possibilities for selecting the potential biomarkers of *W. cocos*. Comprehensively considered, it is necessary that triterpenoids can be used for screening the source range of potential biomarkers by network pharmacology on *W. cocos*. However, only screening biological markers is not enough from the perspective of effectiveness, it is also necessary to explore the different components between *Poria* and *Poriae* cutis from the perspective of measurability.

It is necessary to consider the characteristics of TCM, such as multi-component, multi-target, integrity, and others. The effective scientific characterization with modern technologies was used in TCM evaluation,which is an innovative, forwardlooking, and applicable new model. For example, chemical fingerprint technology, as a comprehensive and quantifiable analysis method, can reflect the active components contained in substances and effectively characterize the quality of TCM's materials (Zhou et al., 2015). Chromatography is a common method, especially high-performance liquid chromatography (HPLC), gas chromatograph, gas chromatography-mass spectrometry, liquid chromatography– mass spectrometry, etc., all of which have become accepted as routine analytical technologies (Seger et al., 2013; Ma et al., 2019; Yue et al., 2021b). HPLC has the characteristics of high separation efficiency, high selectivity, high detection sensitivity, fast analysis speed, and a wide application range. Even though several other qualitative chromatographies can carry out an accurate quantitative analysis of chemical components, it has the disadvantages of complex sample processing, high professional requirements, and high cost (Sarkar et al., 2021; Wang et al., 2021). HPLC is the preferred method of TCM fingerprint technology. However, multi-component synergy is an important aspect of the pharmacological activity of TCM. This means that it is not possible to evaluate the overall quality of the components only by considering their content. Molecular spectroscopy, particularly vibrational spectroscopy and electronic spectroscopy, has been used extensively in a wide range of areas of chemical sciences, material science, and nanoscience- and biosciences, as well as medicinal plant quality control due to it providing valuable information about the structure, reactions of molecules, and function. One can use anharmonic approaches and grid-based approaches for both infrared and near-infrared (NIR) spectroscopies (Bec et al., 2020; Ozaki et al., 2021). Compared with HPLC, Fourier transform near-infrared spectroscopy (FT-NIR) has the advantages such as being low cost, a green technology, non-destructive, and fast, which is widely used in the food industry, drug manufacturing, agricultural production, and other fields (Li et al., 2018; He et al., 2020; Zhang et al., 2021). Chromatography and spectrometry combined with machine learning are the commonly used technology for the quality evaluation of *W. cocos* (Liu et al., 2019; Wang et al., 2019).

The continuous development and advancement of information technology and biotechnology supply the data for pharmacological investigation and application. It is difficult to make full use of large-scale data with simple statistical analysis methods. To improve the efficiency of analysis and keep up with the development of the times, the currently hotly discussed artificial intelligence technology should be considered for quality evaluation of TMC. Machine learning is a common research hotspot in the field of artificial intelligence and pattern recognition, which is widely used in different fields of food technology (Sarkar et al., 2022a,b), medicinal plants, and fungi (Li et al., 2022). Common algorithms include deep learning, support vector machines, partial least squares discriminant analysis (PLS-DA), etc. (Liu et al., 2020; Dong et al., 2021; Li et al., 2022a). Deep learning is the main method in artificial intelligence research and development at this stage. It has excellent advantages in image classification and object recognition. Combining deep learning with two-dimensional correlation spectral imaging for the evaluation of TCM can give full play to the respective advantages of the two technologies, which greatly improves the analysis efficiency and promotes the modernization of TCM (Dong et al., 2020). The information obtained *via* deep learning in the learning process, including text, image, and sound data interpretation, has made a great contribution. Convolutional neural network (CNN), as a neural network system based on convolution operation, is one of the deep learning techniques. In theory, the network results in a new model by adding new layers so that it might better fit the training dataset, so adding layers seems easier to reduce training error. However, the training error is also increased after adding too many layers. In response to this problem, the residual network (ResNet) was proposed by He Kaiming' team in 2015. ResNet won three championships in image classification, detection, and localization in the ILSVRC (Russakovsky et al., 2014). Its main contribution is the discovery of “degradation” and the invention of “Quick connection” for the degradation phenomenon. The “shortcut connection” or “skip connection” were discovered against the phenomenon of “degradation,” which greatly eliminates the difficulty of training neural networks with excessive depth (He et al., 2015). In previous research, ResNet combined with chemometrics had applications in qualitative and quantitative aspects of plants, fungi, etc. (Ding et al., 2021; Liu et al., 2021; Wang et al., 2021; Yue et al., 2021a). However, there are rare literature reports on machine learning and network pharmacology based on potently non-destructive FT-NIR and HPLC to screen the potential biomarkers for different parts of *W. cocos*, which may contribute to rational medicine use and more product development. Therefore, from the perspective of measurability and effectiveness, we combined machine learning and network pharmacology methods to further explore the chemical components differences between the *Poria* and *Poriae* cutis of *W. cocos*, which may provide scientific evidence for its traditional clinical experience application.

**Figure 1:**
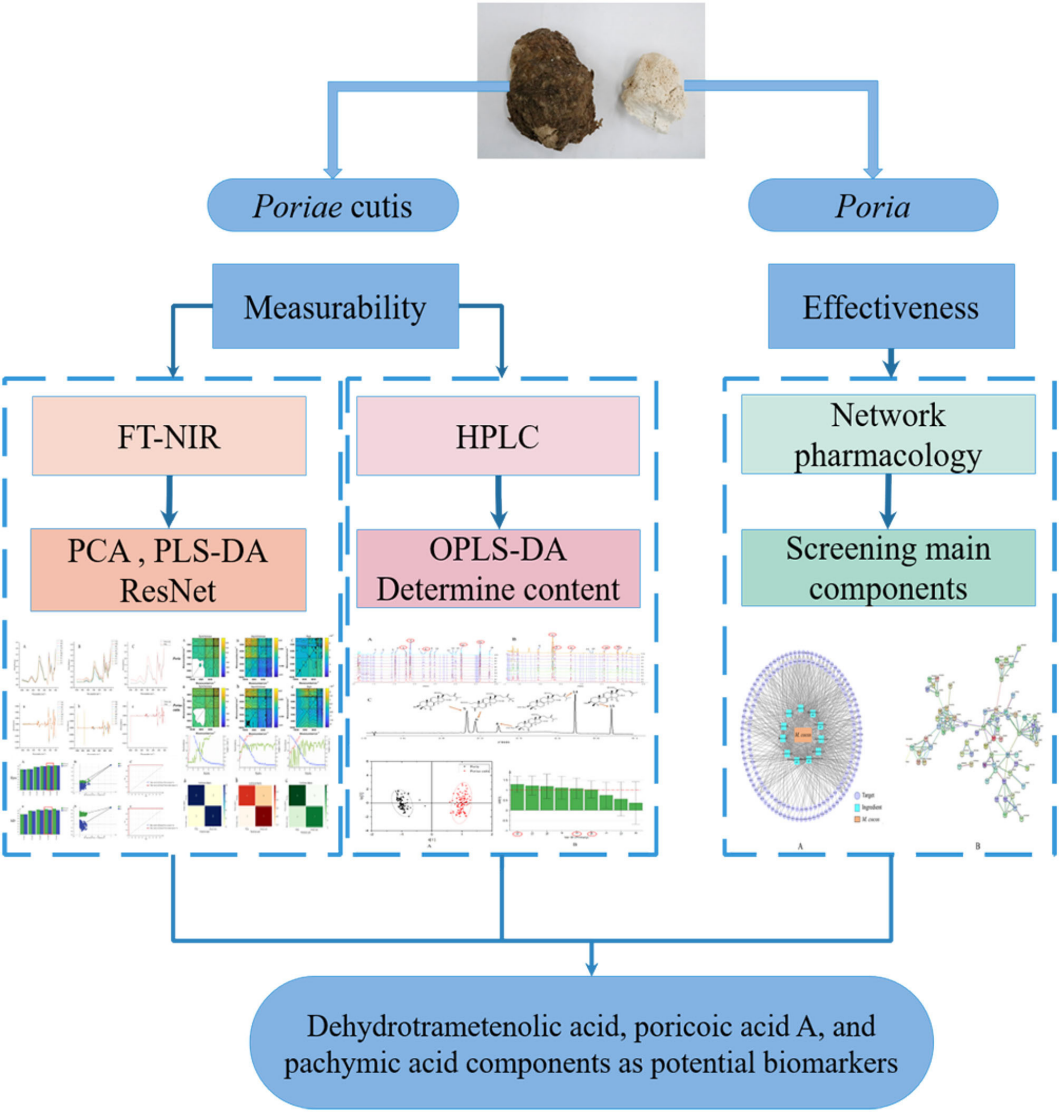
The flow chart of the full text.

The objectives of this study were (1) to establish a qualitative (*via* PCA, PLS-DA, ResNet, and OPLS-DA) strategy for distinguishing the *Poria* and *Poriae* cutis samples based on the FT-NIR spectrum and HPLC fingerprint; (2) to screen the potential biomarkers for classifying *Poria* and *Poriae* cutis samples with network pharmacology and HPLC quantitative analysis. The flow chat of the full text is shown in Figure 1. First of all, the FT-NIR spectrum of *Poria* and *Poriae* cutis samples was collected. Then, qualitative analysis was carried out based on multivariate data analysis of the FT-NIR spectra. HPLC was used to generate reference analytical data for the major triterpene components. Combined with network pharmacology, the potential biomarkers were screened for *Poria* and *Poriae* cutis. Finally, the proposed strategy might provide a rapid, practical, and comprehensive method to evaluate and distinguish different parts of medicinal and edible fungi such as *W. cocos*.

## Materials and Methods

### Materials and Samples

A total of 200 samples were collected from 10 different regions for *Poria* and *Poriae* cutis of *W. cocos* in Yunnan Province, China. It was identified as *Macrohyporia coco*s (Schwein.) I. Johans. & Ryvarden by associate researcher YuanZhong Wang from the Institute of Medicinal Plants, Yunnan Academy of Agricultural Sciences. After sample collection, we cleaned the soil on the surface and placed it in a cool and ventilated place to air-dry. During this process, these samples were turned over in time. When the water was almost evaporated for whole *W. cocos*, the *Poria* and *Poriae* cutis were separated, pulverized with the pulverizer for each sample, passed by 60-mesh sieves, stored in a self-sealing bag, and placed in a dry and sealed environment to save for further use.

### Acquire the FT-NIR With Different Parts of *W. cocos*

Fourier transform near-infrared spectra were gathered using a diffuse reflectance microscope by an Antaris II Fourier transform near-infrared spectrometer. Approximately 10 g of powder was weighed and placed in a glass vessel for compression and scanning for each sample. The scanning wavenumber range is 10,000–4,000 cm^−1^, the cumulative number of signal scans is 64, and the subresolution is 4 cm^−1^. Each sample was scanned three times, and the average spectrum was finally obtained by OMNIC 9.7.7 software for chemometric analysis.

### Spectral Data Processing

The unprocessed spectral data have problems such as baseline drift and noise interference, which make the characteristic absorption peaks in the spectrum covered, and the spectral resolution is reduced, which affects the further analysis of the spectral data. To improve the spectral resolution, eliminate the influence of the above factors, and reflect the characteristics of FT-NIR to the greatest extent, it is necessary to preprocess the original data. Preprocessing is the need to correct for baseline changes, scatter, noise, peak shifts, and missing values (Rinnan et al., 2009; Engel et al., 2013; Lee et al., 2017). There are several preprocessing technologies available here, including multiplicative scatter correction (MSC) and their several variants, standard normal variate (SNV), and second derivative (SD) (Roger et al., 2020). Considering that the SD method can effectively remove the wavelength linearly related drift, the resolution of the fine-grained absorption peaks generated by the fine-grained structure in the original spectrum is improved, as well as its wide application and superiority in spectral preprocessing. Therefore, we compared the preprocessing effect of the SD on FT-NIR spectra and the spectra without SD preprocessing data in this study. Before modeling, the spectral data of each part are divided into 2/3 (67) training set and 1/3 (33) test set using the Kennard–Stone algorithm for establishing the PLS-DA model further.

### HPLC Analysis of Triterpenoids in Different *Poria* and *Poria* Cutis Samples

A powder sample analysis was performed with Agilent 1290 Infinity II HPLC system (Agilent Technologies, USA) that is composed of a G7117B diode-array detector (UV, DAD, 190–400 nm), a G7116 thermostatted column compartment, a G7120A binary gradient pump, and a G7167B multi-sampler auto-sampler. All samples were selected from each part (*Poria*: 30; *Poriae* cutis: 30) for HPLC fingerprint analysis and 60 samples were randomly selected for content determination (poricoic acid A, pachymic acid, and dehydrotrametenolic acid). About 0.5 mg of each sample powder was precisely weighed and put in a stoppered test tube, and 2.0 ml of methanol solution was added, extracted by ultrasonic for 40 min at the power of 150 W and working frequency of 55 kHz. After cooling to room temperature, it was filtered with a 0.22-μm needle filter and the test solution was obtained for the next analysis. Five reference substances were precisely weighed and methanol was added to obtain standard solutions for subsequent determinations. HPLC chromatographic conditions with the Inertsil ODS-HLHP column (3.0 × 150 mm, 3 μm) were set as follows: the flow rate was 0.4 ml·min^−1^; the column temperature was 40°C; the detection wavelengths were 242 nm; and the injection volume was 7 μl. The mobile phase was 0.1% aqueous acid (A) and acetonitrile (B), and each sample was eluted according to the following gradient: 40% B (0.00 → 25.00 min), 40 → 69% B (25.00 → 52.00 min), 69 → 72% B (52.00 → 56.00 min), 72 → 78% B (56.00 → 58.00 min), 78 → 90% B (58.00 → 58.01 min), and 90% B (58.01 → 60 min). In addition, methodological verification was performed in this process so as to ensure accurate and reliable analysis results, such as limit of detections (LODs), limit of quantifications (LOQs), and recovery rate of stability, precision, and repeatability. Among them, the LOD and the LOQ were equal to a signal-to-noise of 3 and 10, respectively. For inter-day precision, the sample was analyzed six times (0, 6, 12, 17, 21, and 24) each day and for over 3 consecutive days for intra-day precision. The recovery rate was analyzed using stock solutions spiked with low, medium, and high concentrations.

### Chemometrics Analysis Based on Spectral Data

Qualitative analysis was executed using multivariate statistical analysis methods including PCA, PLS-DA, and ResNet based on FT-NIR spectral data in this study. In addition, the OPLS-DA model was established by SIMCA P14+ for the relative peak area of the common peaks between *Poria* and *Poriae* cutis, and the important variables were filtered by VIP > 1 to evaluate the important difference components between the *Poria* and *Poriae* cutis.

### PCA

The principal component analysis (PCA), as a common data analysis method, is often used for dimensionality reduction of high-dimensional data to extract the main feature components of the data, which can quickly identify the differences between samples and allow the visualization of classes and outliers (Zhou et al., 2020). A set of potentially correlated variables was transformed into a set of linearly uncorrelated variables through orthogonal transformation, and the transformed set of variables is called principal components. PCA was used to delete the redundant variables (closely related variables) for all the originally proposed variables and establish a few new variables as possible. These new variables were uncorrelated, but the informational aspects of the study were kept as original as possible. In this study, the FT-NIR data of *Poria* and *Poriae* cutis were imported into SIMCA P14+ software to obtain PCA score plots (PC1 and PC2) and explore the regularities of distribution for all different parts of samples.

### PLS-DA

Partial least squares discriminant analysis (PLS-DA) is a statistical method related to principal components. After the data are dimensionally reduced, a regression model is established and the results are discriminant analysis. Model parameters include Y-axis cumulative interpretation rate (R^2^Y), model cumulative prediction pate (Q^2^), root mean square error of calibration (RMSEC), and cross-validation root mean square error (RMSECV), all of which can reflect a lot of information about variables. The parameters are also related to non-error rate (%NER) and correct discrimination rate (%CCR), which are defined as each class = 100 × (correctly classified)/(total) and all classes = 100 × (correctly classified)/(total), respectively. AUC is the area under the curve of ROC (receiver operating characteristic). What is more, the variable is obtained according to the largest covariance of the classification matrix Y from the variable matrix X. Y is usually divided into two categories, that is, Y = 1 represents the samples of the discriminating class and Y = 0 represents all remaining samples. In this study, the different parts of *W. cocos* were analyzed and identified by the PLS-DA model. A total of three parameters are essential except those mentioned above, such as specificity (SPE), sensitivity (SEN), and accuracy (ACC), which are often used as an evaluation index and reflect the performance of PLS-DA. Their expressions are shown below:


(1) $$\begin{array}{rcl} Sensitivity & = & \frac{TP}{\left(TP+FN\right)} \end{array}$$

(2) $$\begin{array}{rcl} Specificity & = & \frac{TN}{\left(TN+FP\right)} \end{array}$$

(3) $$\begin{array}{rcl} Accuracy & = & \frac{TN+TP}{TN+TP+FP+FN} \end{array}$$

It is noteworthy that the sensitivity, specificity, and accuracy are calculated by means of true positive (TP), true negative (TN), false positive (FP), and false negative (FN). For the parameters of R^2^-intercept and Q^2^-intercepts, 200 iterations were performed by SIMCA P14+ software to examine the robustness and fitting degree of the PLS-DA model.

### Acquisition of Two-Dimensional Correlation Spectroscopy Image

Because traditional Chinese medicine has the characteristics of “multi-component, multi-target, multi-channel” and the signals of different chemical components overlap each other, it is difficult to extract interesting information from one-dimensional spectroscopy. 2DCOS is an effective technology that can resolve overlapping bands and improve spectral resolution by designing perturbations.

Synchronous and asynchronous 2DCOS spectra are calculated using the generalized 2DCOS algorithm. Synthetic 2D correlation spectroscopy is multiplied by synchronous and asynchronous 2DCOS. Φ(*v*_1_, *v*_2_) shows synchronous 2DCOS spectra, Ψ(*v*_1_, *v*_2_) means the synchronous 2DCOS spectra, *N*(*v*_1_, *v*_2_) represents the integrated 2DCOS (i2DCOS), and the formula is as follows:


(4) $$\begin{array}{rcl} \Phi \left(v_{1},\;v_{2}\right) & = & \;\frac{1}{m-1}{W\left(v_{1}\right)}^{T}.W\left(v_{2}\right) \end{array}$$

(5) $$\begin{array}{rcl} \text{Ψ}\left(v_{1},v_{2}\right) & = & \frac{1}{m-1}{W\left(v_{1}\right)}^{T}.W\left(v_{2}\right).N \end{array}$$

(6) $$\begin{array}{rcl} N\left(v_{1},v_{2}\right) & = & \left[\Phi \left(v_{1},\;v_{2}\right)\right].\left[\text{Ψ}\left(v_{1},v_{2}\right)\right], \end{array}$$

where *N* is the Hilbert-Noda matrix, and it is defined as follows:


(7) $$M_{jk}=\{\begin{array}{cc} 0\&j=k & \;\\ \frac{1}{\pi (k-j)}\&j\neq k & \; \end{array}$$

when the spectrum with perturbation interval *t* is measured at order *n*, and the dynamic spectral intensity is represented as a column vector *F* under the variable *v*. The expressions of *F*(*v*) can be defined as follows:


(8) $$F\left(v\right)=\left|\begin{array}{c} \begin{array}{c} f\left(v,t_{1}\right)\\ f\left(v,t_{2}\right) \end{array}\\ \begin{array}{c} f\left(v,t_{3}\right)\\ . \end{array}\\ \begin{array}{c} .\\ .\\ f(v,t_{n}) \end{array} \end{array}\right|$$

About 90% (180 samples) of images were randomly selected for ResNet modeling, and the remaining 10% (20 samples) were used for external verification. The Kennard–Stone algorithm was used to select the training set (80%) and test set (20%) from the 180 samples, and then, MATLAB2017b was used to automatically generate 600 two-dimensional correlation spectra (synchronous 2DCOS: 180; asynchronous 2DCOS: 180; integrated 2DCOS: 180) for further modeling of ResNet.

### ResNet

Convolutional neural network (CNN) is a class of feedforward neural networks that include convolutional computation and have a deep structure. It is one of the representative algorithms of deep learning, which is widely used in many fields, such as speech recognition, machine translation, and so on (Kalchbrenner et al., 2016; Sercu and Goel, 2016). ResNet, as a super CNN network, is easy to optimize, and the accuracy can be improved by increasing the depth. The internal residual block adopts skip connections and can alleviate this problem of the gradient disappearance caused by increasing depth in deep neural networks. In traditional convolutional layers or fully connected layers, there may be some problems such as information loss, yet ResNet can solve this problem to a certain extent (Lu et al., 2018). At the same time, the integrity of the information was protected by directly passing the input information to the output. The operation steps are simplified so that the whole network just needs to understand the difference between input and output. Supposing the input of a neural network is x and the expected output is *H(x), H(x)* is expected to be a complex potential map, but it is more difficult to learn. If we take the input *x* directly as the initial result (*via* shortcut connections), then what we need to learn is *F(x)* = *H(x)*-*x*. So, ResNet converts the learning objective to residuals instead of learning the whole output. The schematic diagram of the residual block is shown in Supplementary Figure S1. The residual convolution neural network model that is used in this study is displayed in Supplementary Figure S2, and the input data are synchronous, asynchronous 2DCOS, or i2DCOS spectral images.

### OPLS-DA

Different from the PCA method, OPLS-DA is a supervised discriminant analysis statistical method. This method uses PLS-DA to establish the relationship model between metabolite expression and sample category to realize the prediction of sample category (Fang et al., 2013). Among them, R^2^X and R^2^Y represent the interpretation rate of the X and Y matrices of the built model, respectively. Q^2^ indicates the predictive ability of the model. In theory, the closer the values of R^2^ and Q^2^ are to 1, the better the model, and the lower the value, the better the model and the worse the fitting accuracy of the model. Usually, it is better for R^2^ and Q^2^ to be higher than 0.5 (50%), but higher than 0.4 is acceptable, and the difference between the two should not be too large. At the same time, the VIP is calculated to measure the impact strength and explanatory power of each chemical component on the classification and discrimination of each group of samples so as to assist the screening of marker metabolites (usually VIP value > 1.0 as filter criteria). The relative peak area of the common peaks of 200 samples of *Poria* and *Poriae* cutis was obtained by SIMCA P14+ software, and the relative peak area of 9 common peaks was used as a variable to establish an OPLS-DA model.

### Screening of Potential Biomarkers to Distinguish *Poria* and *Poria* Cutis Sample Structures by the Network Pharmacology

Active components and targets of *W. cocos* were screened in the TCMSP database (http://tcmspw.com/tcmsp.php) and in literature, and oral bioavailability (OB) ≥ 30% and drug-like properties (DL) ≥ 0.18 were used as the screening conditions. The collected active component was entered into Pubchem (https://pubchem.ncbi.nlm.nih.gov/), and the corresponding molecular structures were searched. Canonical simplified molecular-input line-entry system (SMILES) was recorded and put into the Swiss target prediction (www.SwissTargetPrediction.ch) database to obtain each predicted target of components. Then, an “Active-ingredient-target” network was constructed by Cytoscape3.7.1 soft as the background and prediction target as the interest gene. At the same time, target prediction and protein interaction (PPI) were selected to analyze the network topology and select parameters greater than the median and degree ≥ 6 as the potential core target. Using the David 6.8 database (https://david.ncifcrf.gov/), the functional enrichment analysis and visualization (gene ontology, GO and Kyoto Encyclopedia of Genes and Genomes, KEGG) of 20 potential core target proteins were performed, and the conditions of *p-value* < *0.05* were used for screening. Finally, a network “component-target-pathway” was visualized using Cytoscape3.7.1 software to initially screen for potential markers of *W. cocos*.

### Analysis of Triterpenoid Content

A total of 60 samples randomly from the same origin of different parts of *W. cocos* were mixed in the same proportion to determine the content of triterpenoids (dehydrotumulosic acid, poricoic acid A, dehydropachymic acid, pachymic acid, and dehydrotrametenolic acid). The five reference solutions were prepared with methanol to the concentrations of 5.00–999 μg·ml^−1^ for dehydrotumulosic acid , 0.22–6,730 μg·ml^−1^ for poricoic acid A, 2.4–480 μg·ml^−1^ for dehydropachymic acid, 10.3–1,240 μg·ml^−1^ for pachymic acid, and 0.49–2,450 μg·ml^−1^ for dehydrotrametenolic acid. The reference solutions were determined according to the chromatographic conditions under section Spectral Data Processing. By taking the peak area value Y as the ordinate and the reference substance mass concentration X as the abscissa, we drew a standard curve and calculated the regression equation. Finally, the contents of three components in different parts of *W. cocos* were calculated and compared, and the components with large differences in content are preliminarily screened out as potential markers.

## Result and Discussion

### Analysis of FT-NIR Spectra

**Figure 2:**
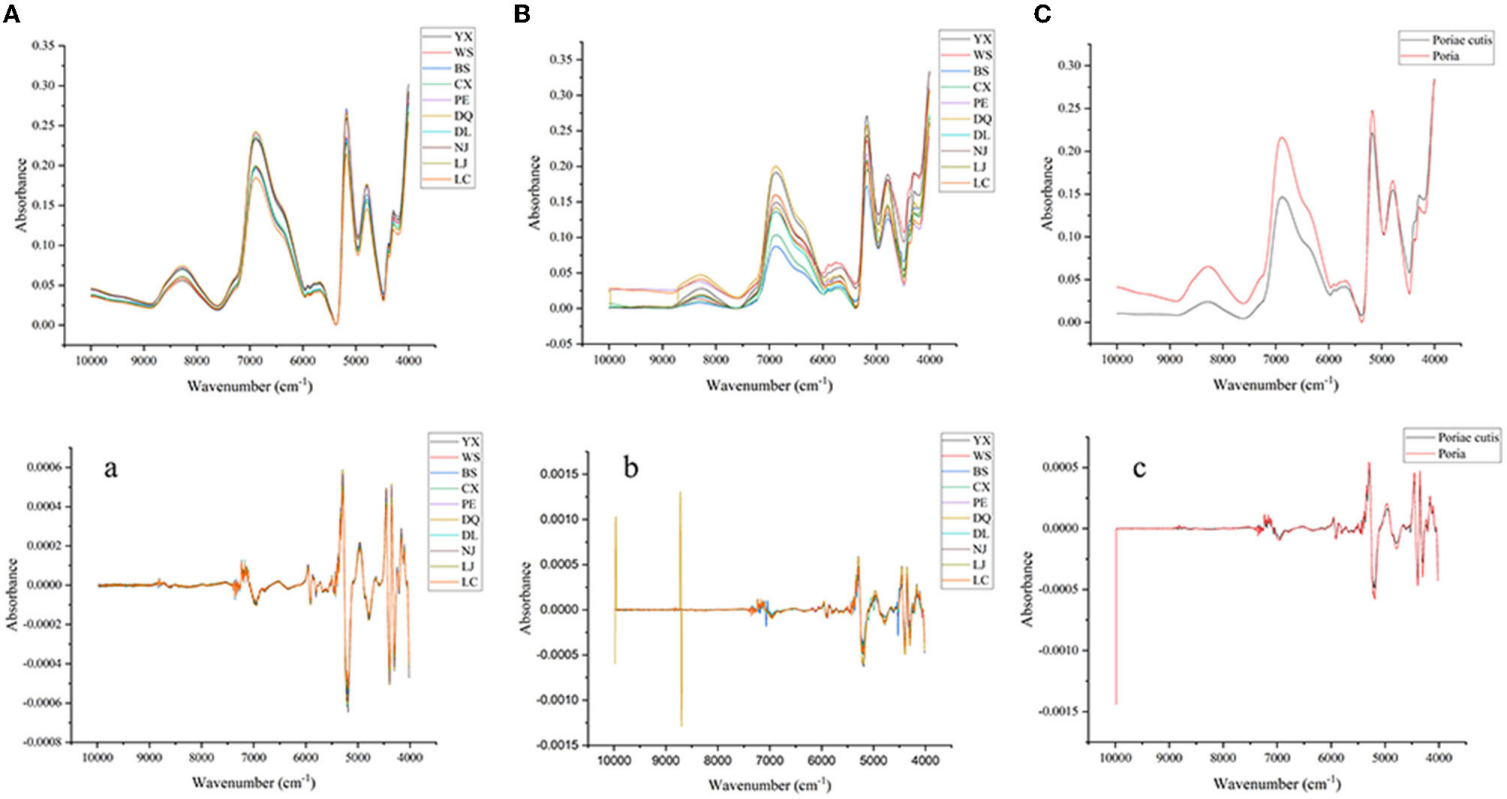
Averaged raw and SD spectra of *W. cocos*. **(A** and **a)** Different regions of *Poria*; **(B** and **b)** Different regions of *Poriae* cutis; **(C** and **c)** Different parts.

Fourier transform near-infrared (FT-NIR) spectroscopy is an attractive technology for the food or TCM industry because it can characterize chemical information in a simple, fast, and non-destructive manner. The main reason is the non-resonance of molecular vibration, which is generated when the molecular vibration transitions from the ground state to the high energy level and have a strong penetration ability. In this study, the FT-NIR of all samples can be seen as the accumulation of their components, thus maintaining the integrity of the TCM. At the same time, the FT-NIR exhibits that the main components in the mixture existed in the form of characteristic absorption bands. The components with high content indicate more characteristic absorption bands, yet the components with low content demonstrate less characteristic absorption bands, and some characteristic peaks would be overlapped. Figures 2A–C are the raw average spectra of *Poria* and *Poriae* cutis from different origins based on FT-NIR spectra. These different peaks represented the vibration mode and the base group of the *W. cocos* samples and their attribution. Figures 2A,B showed that the peak shapes for different parts of *W. cocos* from different regions had similar trends and showed no obvious difference in absorbance, which means that the chemical component information between different origins was similar. Compared with *Poria*, the absorption peak intensity of different origins was significantly different based on *Poriae* cutis, especially the absorption peak in the band of 7,200–5,350 and 5,200–4,000 cm^−1^. It could be seen from the average spectrum of different parts (Figure 2C) that there were significant differences in peak shape and peak position mainly in the three wavenumber ranges of 8,850–7,750, 7,250–6,000, and 4,450–4,050 cm^−1^, but the difference in absorbance was obvious. Figure 2C shows that the chemical components in different parts of *W. cocos* are similar but the content of the components is different. There were double frequency peaks and fundamental frequency peaks of C-H at 8,300 and 4,254–4,377 cm^−1^, which were related to components such as polysaccharides and water in *W. cocos* (Zhao et al., 2014). The stretching vibration around 6850 and 5,560–5,730 cm^−1^ was related to the C-H functional group (Zhou et al., 2006). In addition, 5,200, 4,450–4,400, and 4,100 cm^−1^ were the stretching vibrations of functional groups including O-H, O-H, and C-O, respectively (Alamprese et al., 2013). Figures 2a–c show the SD FT-NIR spectra. They also showed more subtle absorption peaks than raw spectra, which provided a feasible basis for the establishment of PCA and PLS-DA based on the SD spectra for the comparison and analysis of different parts.

According to the above analysis, there were indeed significant differences in the distribution and intensities of the peaks for different parts and regions, which also reflected the difference in the relative content of the characteristic components. However, the differences in absorption intensity and peak shape in different regions were much lower than in different parts, which demonstrated that the differences between individuals may be lower than the differences within individuals, and it is easier to identify parts than regions based on FT-NIR. Although the FT-NIR spectrum can reflect the subtle differences between samples, the identification of different parts and regions cannot be accurately completed, so further identification and analysis of *W. cocos* are needed to combine with chemometrics.

### HPLC Analysis of Triterpenes in Different *Poria* and *Poria* Cutis Samples

**Figure 3:**
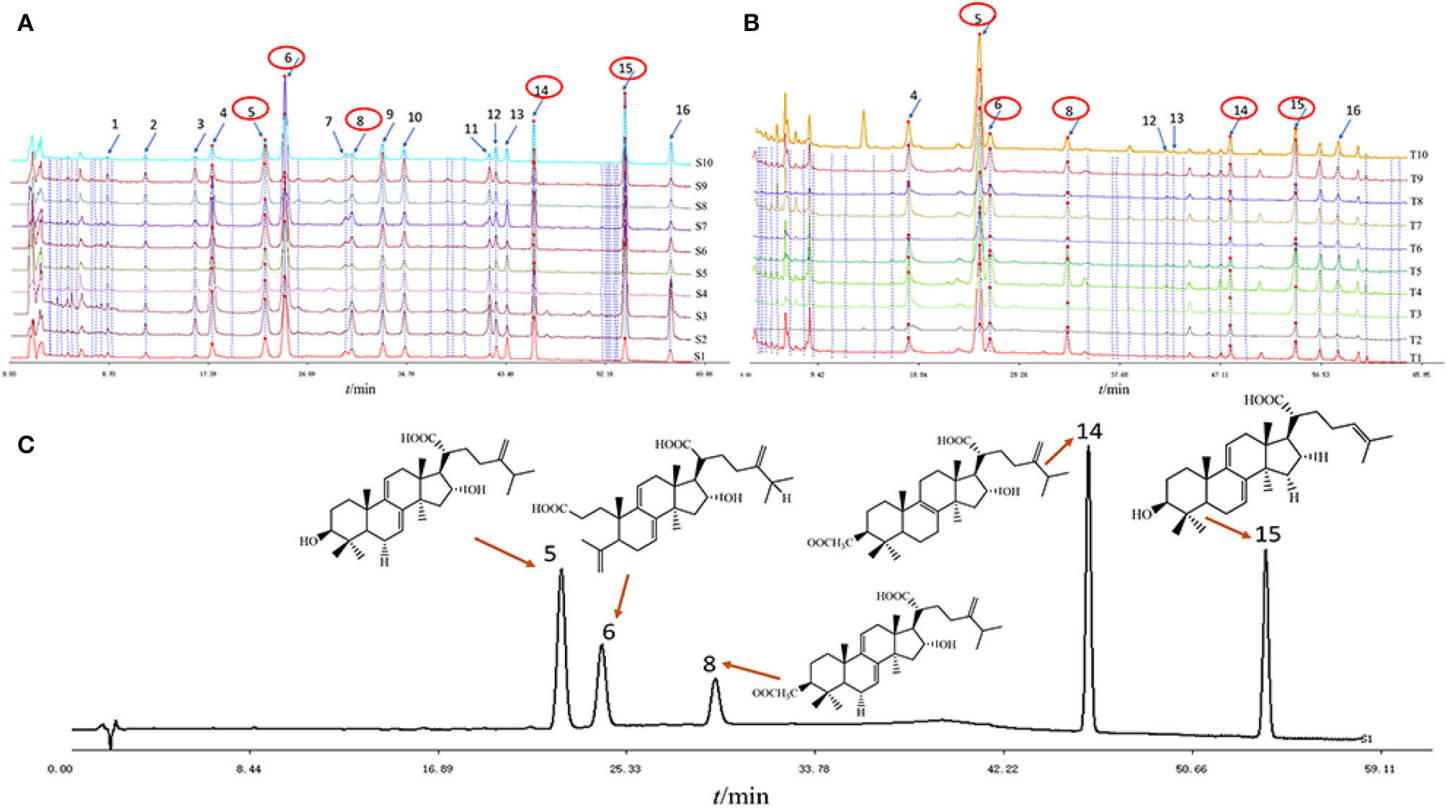
The average chromatograms of the 10 regions of *W. cocos* samples with different parts: **(A)** *Poria*; **(B)** *Poriae* cutis; **(C)** five mixed reference substances.

High-performance liquid chromatography (HPLC) has the advantages of high accuracy, high sensitivity, good qualitative, and quantitative, among others, which has been widely used in the field of food chemical industry and TCM. Based on the results of section Analysis of FT-NIR Spectra, the differences between different regions were ignored due to fewer variations between samples compared to variations in different parts and all samples from each part of *W. cocos* for further research. In this study, five chemical components were determined by HPLC due to triterpenes having attracted the attention of researchers because of their extensive pharmacological activities. As seen in Figure 3, HPLC displays the average chromatograms (one average spectrum for each region) of the two parts of the *W. cocos* samples. The obvious components were separated by gradient elution of HPLC, and the signal value of peaks 6 and 15 was much higher than that of other peaks for *Poria* (Figure 3A). From Figure 3B, the signal value of peak 5 was much higher than other peaks for *Poria* cutis. By comparing the retention times of the corresponding target components, these 5 peaks were successfully identified, including peak 5 (dehydrotumulosic acid), peak 6 (poricoic acid A), peak 8 (dehydropachymic acid), peak14 (pachymic acid), and peak 15 (dehydrotrametenolic acid). Overall, the precision, repeatability, stability, and accuracy of the results demonstrate that this analytical method is feasible and credible for quantitative analysis. For more accurate results, all samples were extracted and analyzed on the same day. The parameter results are as follows: correlation coefficients (R^2^) are all >0.99; precision: relative peak area RSD <5.68%; repeatability: relative peak area RSD <5.95%; stability: relative peak area RSD <0.71%; and average recovery rate between 96.32 and 106.4% (compliant with pharmacopeia standards: 85–110%). Combining these method validations, regression equations, linearity ranges, correlation coefficients, LODs, and LOQs of five target compounds were given, and it is shown that this method is feasible to substantiate the robustness of the study with detailed results in the Supplementary Table S1.

## Chemometrica Analysis Based on Spectral Data

### Result of PCA

Principal component analysis (PCA) is a multivariate statistical method. A set of potentially correlated variables is transformed into a set of linearly uncorrelated variables through orthogonal transformation, and the transformed set of variables is called principal components (Miaw et al., 2018). In this study, PCA was also executed to analyze the FT-NIR dataset of *Poria* and *Poriae* cutis samples to discriminate the dissimilarities and similarities between the samples and variables. As seen in Supplementary Figure S3, the PCA score plot based on FT-NIR explains 98.3% of the total variance by two PCs, such as PC1 (56.6%) and PC2 (41.7%). The results showed that the *Poria* and *Poriae* cutis had a good separation effect according to two different categories, which revealed that there were significant differences in chemical components or content between samples of the two categories. Regrettably, two samples of *Poria* were separated into the *Poriae* cutis category. The overlapping of two samples may be due to the *Poria* content in these samples approaching the *Poriae* cutis threshold. The other reason might be related to the fact that only some key information about the basic variable structure concerning a potential possibility of separation of *Poria* and *Poriae* cutis samples was presented by PCA (Kimuli et al., 2018). Overall, PCA was able to roughly distinguish *Poria* and *Poriae* cutis, so supervised discriminant analysis was further adopted to improve the separation effect. This result is consistent with section Analysis of FT-NIR Spectra, which can lay a foundation for the subsequent analysis of samples from different parts of *W. cocos*.

### Result of PLS-DA

The performance of a good model was mentioned in the data analysis above, including RMSEE, RMSECV, LVs, Q^2^, specificity, sensitivity, and accuracy. The calculation result in Table 1 presents two preprocessing methods based on different parts of *W. cocos*. Each of them had an excellent ability to distinguish the parts of *W. cocos*. The cumulative contribution rate of the first 5 LVs in the raw dataset Q^2^, R^2^, RMSEE, RMSECV, total train, and test of ACC was 0.928, 0.944, 0.12895, 0.13699, 99.26, and 98.48, respectively. The model based on SD FT-NIR showed the same trend that possessed lower parameters of RMSEE and RMSECV and a high value of Q^2^, R^2^, and total accuracy. The SD model showed similar results to this model based on raw data, and the accuracy of train and test sets was 100 and 96.97%, respectively. It shows that the data preprocessed by SD were not suitable for this set of data, and the effect of the model was not significantly improved. Moreover, the classification parameters of PLS-DA were established by raw and SD FT-NIR, as seen in Table 2. All parameter values were above 0.90, including the train and test sets of SNE, SPE, and ACC. These results indicated that the PLS-DA method was able to efficiently and accurately identify the *W. cocos* with different parts, which also implied that the different parts could affect the chemical properties of *W. cocos*. Both models of raw and SD TF-NIR had good parameters (LVs was 4), which could be used to identify different parts of *W. cocos*. Furthermore, the 200-iteration permutation test performed is shown in Supplementary Figures S4A, 4a, and the right R^2^ and Q^2^ values of the raw and SD data for the permutation test plots of *W. cocos* were higher than the left values. The Y-axis shows the values of R^2^ and Q^2^ in the permutation test plot, and the X-axis represents the similarity between the permutation test data (simulated value) and the original model. The rightmost is the real value, and the left is the analog value. The criteria for judging the validity of the permutation test are as follows: all the blue Q^2^ values on the left are lower than the origin on the right, and the blue regression line of the Q^2^ point and the left vertical axis intersect at zero or less than zero. The results showed that thePLS-DA models were robust and fitting, which was regarded as a model suitable for identifying the different parts of *W. cocos*. To further explore the discrimination performance of the PLS-DA model for each part, AUC, the best latent variable, and the confusion matrix for each class were calculated and plotted, as seen in Supplementary Figure S4, Supplementary Table S2. As a result, the same trend that the AUC value of *Poria* and *Poriae* cutis samples was 1.00 was observed, which could also be found in the ROC plot in Supplementary Figures S4C, S4c. From the confusion matrix in Supplementary Table S2, a total of two samples were wrongly classified, yet the NER% value was more than 96% for the train and test sets of the raw dataset. In the test set of the SD dataset, two samples of *Poria* were wrongly classified as *Poriae* cutis. This might have outliers, consistent with the result that emerged in the exploratory analysis of PCA. In general, the PLS-DA model was established by SIMCA-P14+ based on the raw and SD FT-NIR datasets that can be applied to identify different parts of *W. cocos*. It revealed that there were differences between samples from different parts, which may be caused by the differences in components or component contents in the *Poria* and *Poriae* cutis.

**Table 1:** Parameters for PLS-DA models in part discrimination based on raw and SD FT-NIR datasets.

**Data**	**LVs**	***R* ^2^**	***Q* ^2^**	**RMSEE**	**RMSECV**	**Total accuracy (%)**	
						Train set	Test set
Raw	5	0.944	0.928	0.12895	0.13699	99.26	98.48
SD	4	0.953	0.922	0.08953	0.14558	100	96.97

**Table 2:** The classification parameters of PLS-DA established by raw and SD FT-NIR datesets.

		**Train set**			**Test set**		
	**Classes**	**SNE**	**SPE**	**ACC**	**SNE**	**SPE**	**ACC**
Raw	*Poria*	0.985	1.00	0.993	1.00	0.985	1.00
	*Poriae* cutis	1.00	0.970	0.985	0.970	1.00	0.985
SD	*Poria*	1.00	1.00	1.00	1.00	1.00	1.00
	*Poriae* cutis	1.00	0.939	0.970	0.939	1.00	0.970

### 2DCOS Spectrum Analysis and ResNet

**Figure 4:**
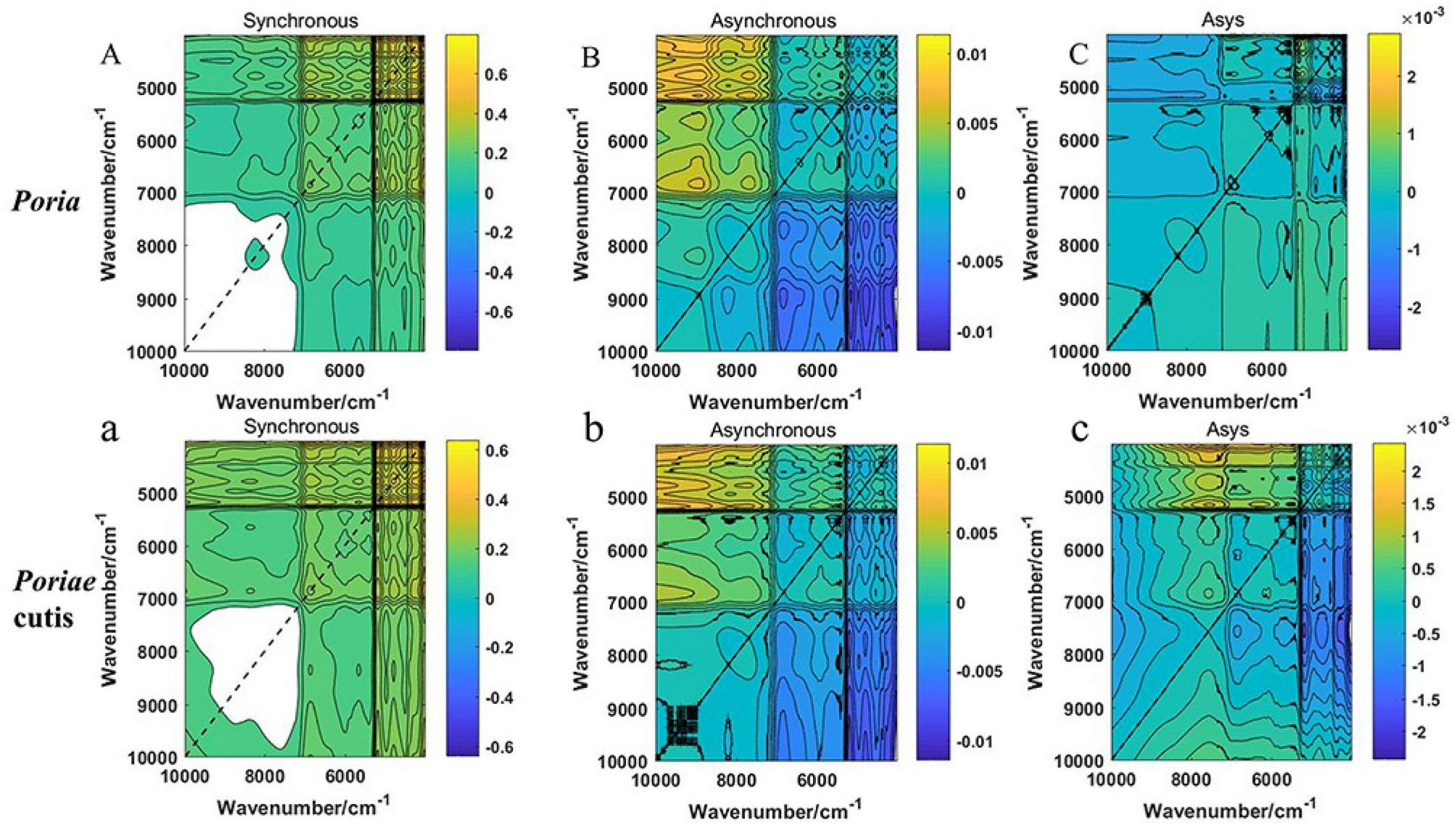
**(A–C)** Synchronous, asynchronous 2DCOS and i2DCOS of W.cocos in Poria; **(a–c)** Synchronous, asynchronous 2DCOS and i2DCOS of W.cocos in Poriae cutis.

Based on the PLS-DA model result, the results of the preprocessed and unprocessed FT-NIR data were similar. Therefore, the raw spectrum of *W. cocos* and the spectrum of 10,001–4,000 cm^−1^ were established for analysis. All 600 2DCOS images were obtained by MATLAB software. The 2DCOS spectrum was shown in Figure 4, including synchronous, asynchronous 2DCOS, and i2DCOS images. From Figures 4A, 4a, the synchronous 2DCOS spectrum takes the diagonal as the axis of symmetry and the correlation peaks appearing on the diagonal are called auto-peaks, which represent the sensitivity of the correlation spectrum to changes in different parts. The peaks on both sides of the diagonal line are called cross-peaks and represent the synchronous changes of peaks at different wavenumbers. Auto-peaking is always positive, but cross-peaking can be positive or negative. If two peaks of different wavenumbers increase or decrease at the same time, the cross-peak on the two-dimensional spectrum is positive; otherwise, it is negative. Figures 4B, 4b were the asynchronous 2DCOS images to characterize the degree of difference between the two spectral signals. Figures 4C, 4c were i2DCOS images, and the peaks were more complex for *Poriae* cutis than *Poria*. From the perspective of the full-band spectra, the peak of the *Poria* was clear and the characteristic information was relatively obvious, especially synchronous 2DCOS. Compared with the asynchronous 2DCOS and i2DCOS, it is not difficult to find that the synchronous spectrum is clearer and has more characteristic peaks. These characteristic peaks facilitate the analysis of auto-peak and cross-peak differences and intensity changes.

**Figure 5:**
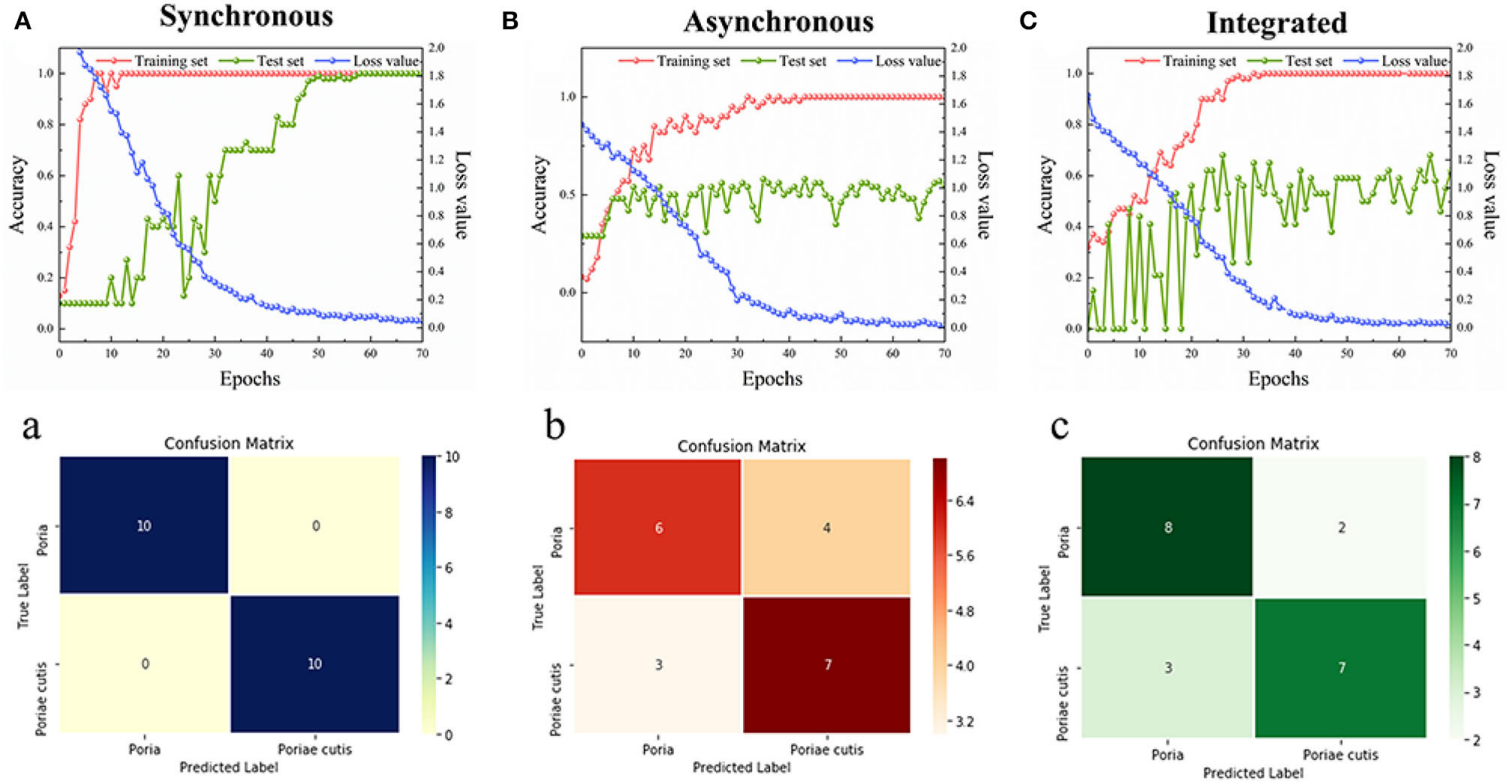
**(A–C)** The accuracy curves and cross-entropy cost function of models based on *W. cocos*; **(a–c)** The confusion matrix for external validation sets of models based on *W. cocos*.

According to the results of the 2DCOS images above, the ResNet model was established by Spyder software to identify the *Poria* and *Poriae* cutis of *W. cocos*. A 12-layer ResNet was constructed, and the model parameters of the learning rate and weight attenuation coefficient λ were 0.01 and 0.0001, respectively. The discriminative performance of the model was determined by the loss and accuracy values. The loss value of the established model was close to zero and the accuracy values were close to 100%, indicating that the model had better performance. Figures 5a,B,C show double coordinate plots of different parts of *W. cocos* based on the synchronous 2DCOS, asynchronous 2DCOS, and i2DCOS spectral models. The result showed that the accuracy of the training set based on the synchronous 2DCOS model was 100%, the accuracy of the test set was 100%, the epoch was 57, and the loss value was 0.069, as seen in Figure 5a. The accuracy of the training set based on the asynchronous 2DCOS spectral model was 100%, the epoch was 43, and the loss value was 0.025. However, the accuracy of the test set was not 100% when the epoch was 70 in Figure 5b. For Figure 5c, the results of the i2DCOS spectral model were similar to the asynchronous 2DCOS, which were not suitable for identifying different parts of *W. cocos*. In a word, synchronous 2DCOS was able to accurately identify two parts, which was consistent with the above conclusions.

In our study, the confusion matrix of the synchronous 2DCOS, asynchronous 2DCOS, and i2DCOS spectra models above belonging to the external validation set was classified, as displayed in Figures 5a–c. The results were as follows: (1) Figure 5a was the confusion matrix of the synchronous 2DCOS model, indicating that all 20 samples of two parts were discriminated correctly; (2) Figure 5b represented the confusion matrix of the asynchronous 2DCOS spectral model, and seven samples were misclassification whereas the remaining 13 samples were correct classification; (3) Figure 5c represented the i2DCOS spectral model, where two samples of *Poria* were misclassified to *Poriae* cutis, and three samples of *Poriae* cutis were misclassified to *Poria*. Therefore, the synchronous 2DCOS spectral model of *W. cocos* had the strongest generalization ability.

### Result of OPLS-DA

**Figure 6:**
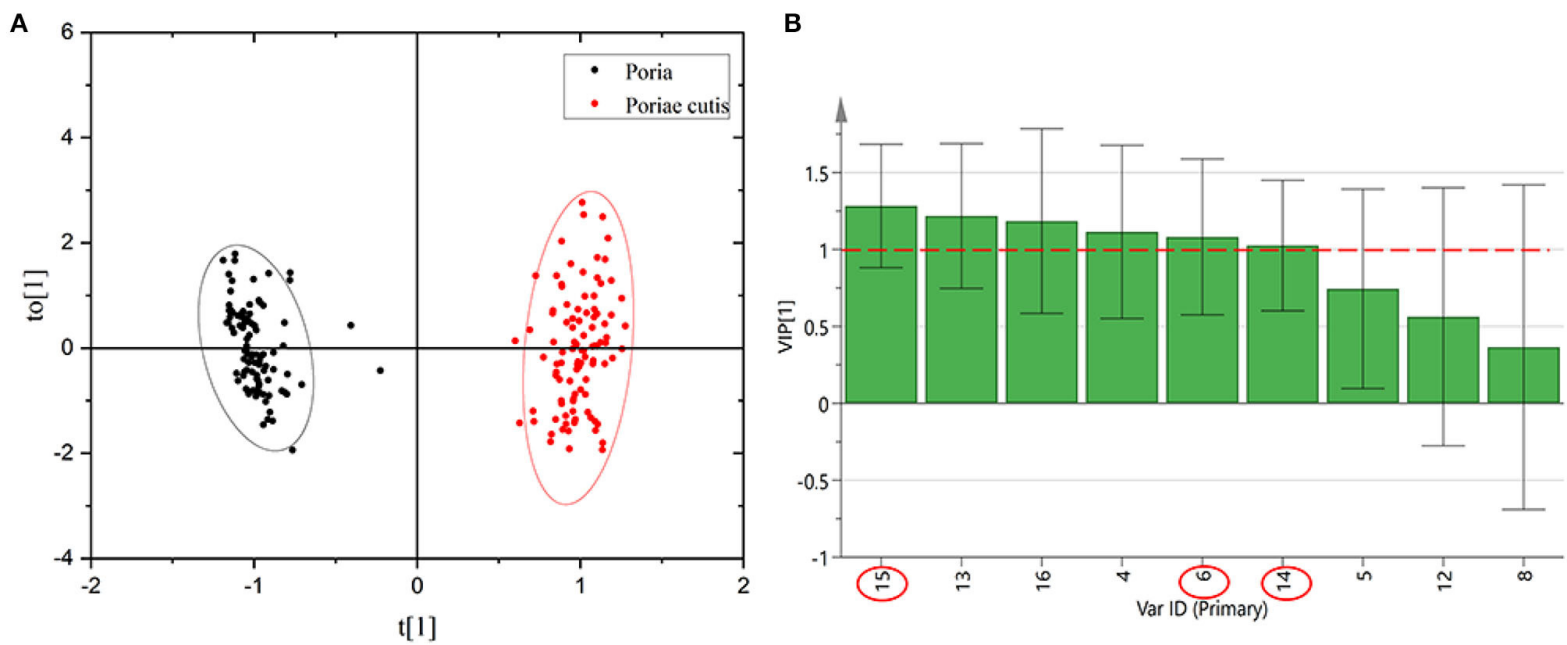
OPLS-DA score scatter diagram **(A)** and common peak VIP value of HPLC **(B)** of *Poria* and *Poriae* cutis.

In view of the fact that the unsupervised qualitative method of PCA clustered the *Poria* and *Poria*e cutis samples. The unknown *W. cocos* samples could not be identified by PCA. Therefore, this study adopted supervised OPLS-DA to analyze and classify the *Poria* and *Poria*e cutis samples based on HPLC data. The relative peak area of the common peaks of *Poria* and *Poriae* cutis was executed *via* SIMCA P14+ software. The relative peak area of the 9 common peaks was used as a variable to establish the OPLS-DA model as seen in Figure 6A. The results were similar to the unsupervised PCA, and all samples were divided into two parts and showed significant differences between the different parts. The model parameter values were as follows: R^2^X = 0.915, R^2^X = 0.949, and Q^2^ = 0.869>0.5, indicating that the classification model was effective. The VIP value was obtained based on this model, as shown in Figure 6B. The larger the VIP value, the farther the variable was from the X-axis, which demonstrated that the chromatographic peaks contributed more to the classification of *Poria* and *Poriae cutis*. It was also the differential component that caused the difference between these two parts. There were 6 peaks with VIP>1, which were 15 (dehydrotrametenolic acid), 13, 16, 4, 6 (poricoic acid A), and 14 (pachymic acid). These components were used to distinguish different origins that play an important role in different parts of *W. cocos* and may be the main marker that lead to the difference in different parts. The value of other peaks was all <1, which was of less significance for distinguishing *Poria* from *Poriae* cutis. Further quantitative analysis of the three components dehydrotrametenolic acid, poricoic acid A, and pachymic acid was carried out.

In conclusion, the qualitative analysis resulted from PCA, PLS-DA, ResNet, and OPLS-DA analysis and showed that simplified FT-NIR and HPLC could provide reliable qualitative results without extensive sample preparation, and they are powerful and practical tools in defining and identifying *Poria* and *Poriae* cutis samples. Based on PCA, PLS-DA, ResNet, and OPLS-DA identification methods, FT-NIR spectroscopy and HPLC would be used as an effective identification for *Poria* and *Poriae* cutis samples.

## Screening of Potential Biomarkers to Distinguish W.Cocos Samples

### Combined With Network Pharmacological Analysis

**Figure 7:**
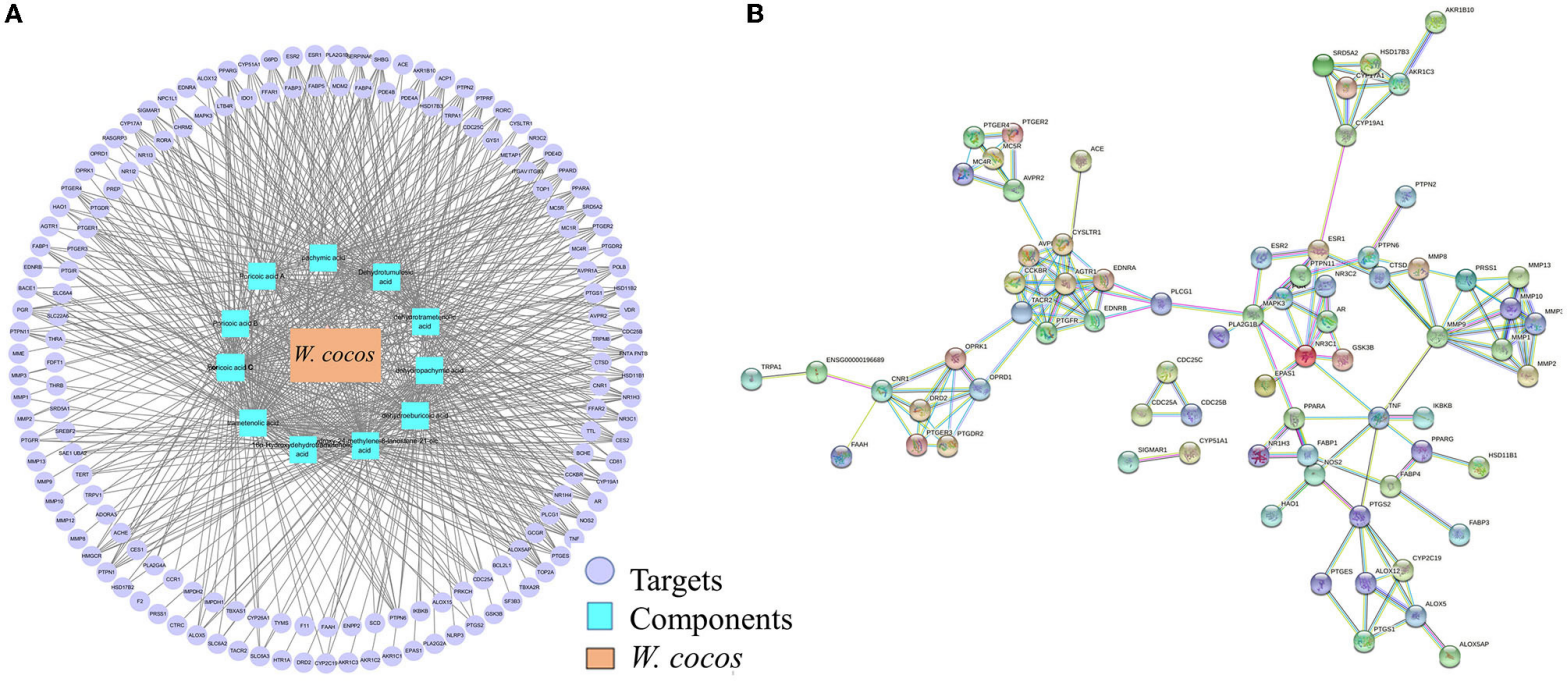
A network pharmacology chart. **(A)** “Active ingredient-target” Network; **(B)** PPI network.

As an empirical system of multi-component therapeutics, herb medicines exert extensive biological and pharmacological effects through multiple components and targets. As screening conditions of oral bioavailability (OB) ≥ 30% and drug-like properties (DL) ≥ 0.18, text mining results indicated 11 active triterpenoids as the potential quality marker candidate active components of *W. cocos*. A total of 160 targets related to the 11 active components were obtained, and the 11 candidate active components and 160 related targets were analyzed by Cytoscape 3.7.1. to build a network diagram of “Active components -target,” as seen in Figure 7A. To screen important core targets, the 160 targets were constructed by Cytoscape 3.7.1 software to build a PPI network (Figure 7B), the highest confidence protein interaction parameter score value was set as > 0.9, but other parameters remainrf unchanged, and a single node in the network was removed. From topological data analysis on the PPI network, we selected parameters greater than the median and degree ≥ 6 as the core target. After screening by this condition, 20 important core targets were acquired, such as AGTR1 (degree = 12), MMP9 (degree = 10), EDNRA (degree = 8), EDNRB (degree = 8), NR3C1 (degree = 8), MAPK3 (degree = 8), AVPR1A (degree = 7), CCKBR (degree = 7), etc. It was found that these targets were mainly related to the components of dehydrotumulosic acid, poricoic acid A, dehydropachymic acid, pachymic acid, dehydrotrametenolic acid, and so on.

The results of functional enrichment analysis and visualization on GO functional analysis and KEGG pathway enrichment analysis are displayed in Supplementary Figure S5, Supplementary Table S3, espectively. GO functional analysis includes cellular component (CC), molecular function (MF), and biological process (BP). Supplementary Figure S5 shows that a total of 19 GO entries were obtained, of which nine were BP, five were MF, and five were CC. The results of the KEGG enrichment analysis are shown in Supplementary Table S3, and the 13 pathways were analyzed according to the *p*-value, indicating that 20 core targets might intervene in the disease to be processed by regulating these signaling pathways and achieving the effect of treating diseases. Based on the above analysis results, the construction of “component-target-pathway” is executed in Supplementary Figure S6. The results showed that 11 components exerted pharmacodynamic effects in different signaling pathways through multiple targets, which was consistent with the characteristics of “multi-component, multi-pathway, and multi-target” in TCM. Using the Cytoscape 3.7.1 software to analyze and taking the “Component-target-pathway” connection degree (degree) as a reference with the site of “Network analysis,” it was found that the degree of connection was higher such as dehydrotermoic acid (degree = 13) and 16α-hydroxyspinene acid (degree = 8), followed by dehydropalicic acid (degree = 6). A total of six targets had higher connectivity of AGTR1 (degree = 12), MMP9 (degree = 10), EDNRA (degree = 8), EDNRB (degree = 8), NR3C1 (degree = 8), and MAPK3 (degree = 8). Furthermore, neuroactive ligand-receptor interaction (degree = 13), calcium signaling pathway (degree = 8), cancer pathway (degree = 7), tumor necrosis factor signaling pathway (degree = 5), and cGMP-PKG signaling pathway (degree = 8) were also higher than other signaling pathways. The above results showed that the 11 main active components of *W. cocos* have played important roles on six important targets and five key signaling pathways. From the perspective of network pharmacological effectiveness, it was observed that triterpenoids such as dehydrotumulosic acid, dehydropachymic acid, pachymic acid, dehydrotrametenolic acid, poricoic acid A, 16α-hydroxydehydrotrametenolic acid, and so on. could be used as the potential biomarkers of *W. cocos* preliminary research on the materials.

### Determination of Triterpenes Content *via* HPLC Analysis

**Figure 8:**
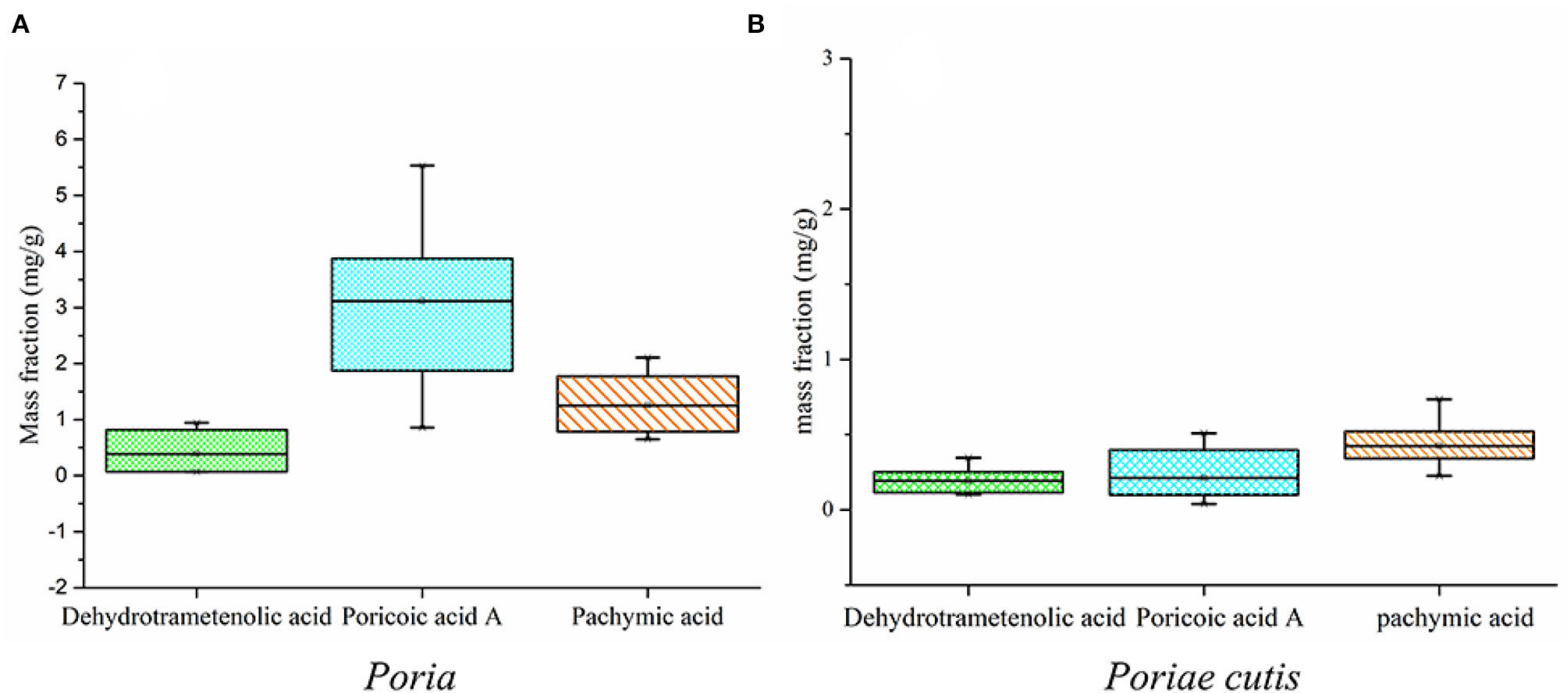
A box plot of dehydrotrametenolic acid, poricoic acid A, and pachymic acid content (%). **(A)** *Poria*; **(B)** *Poriae* cutis.

According to the above qualitative results, the preliminary results showed that the main potential markers of the *W. cocos* sample were dehydrotrametenolic acid, poricoic acid A, and pachymic acid, and the standard curves were Y = 21538210.94X + 107194.87 (R^2^ = 0.9993), Y = 19016331.45X + 1507413.80 (*Poriae* cutis, R^2^ = 0.9998), Y = 22522136.84X + 42388.59 (*Poria*, R^2^ = 0.9999), and Y = 7905709.32X + 42996.45 (R^2^ = 0.9999), respectively. Figure 8 shows the changes in the content of dehydrotrametenolic acid, poricoic acid A, and pachymic acid with the different parts of *W. cocos* and that the content of poricoic acid A and pachymic acid was significantly different, and the change of dehydrotrametenolic acid was not obvious in the two parts of *W. cocos*. The content of poricoic acid A in *Poriae* cutis was higher, followed by pachymic acid and dehydrotrametenolic acid. The changing trend of content for *Poria* was the same as the *Poriae* cutis, but the content of three components was generally higher than that of *Poriae* cutis. This may be why the raw materials of *Poria* are widely used in medical treatment, food, health-care products, etc. In this study, by combining network pharmacology and HPLC quantitative analysis, the perspective of effectiveness and measurability that triterpenoids such as dehydrotrametenolic acid, poricoic acid A, and pachymic acid could be used as the preliminary potential biomarkers of *W. cocos* was demonstrated.

## Integrated Analysis

In fork uses, *Poria* and *Poriae* cutis of *W. cocos* can be used as a potential alternative resource, playing an important role in 14 ethical minorities to treat diseases. Due to growing market demands, long cultivation periods, and consumption of pine trunks during cultivation, the development of alternative methods for the production of *W. cocos* or its active components is of interest. Different parts were carried out based on FT-NIR and HPLC technologies, which could be used as an effective identification and to screen different components between *Poria* and *Poriae* cutis samples. Combined with the network pharmacology analysis, the 11 active components of triterpenoids were screened and targets were predicted. It was revealed that the triterpenoids (such as dehydrotumulosic acid, poricoic acid A, dehydropachymic acid, pachymic acid, dehydrotrametenolic acid, and so on.) possessed these curative effects of anti-tumor, diuretic, anti-inflammatory, and other pharmacodynamic activities by dominating the pathways of tumor pathways, neuroactive ligand–receptor interactions, bladder cancer, and calcium ion signaling to play a role in the treatment of neurological diseases, cancer, and other diseases. *W. cocos* is sweet, light, and flat in nature and returns to the heart, lung, spleen, and kidney meridians. The signaling pathways such as bladder cancer and lung cancer proteoglycans were related to the bladder and lung and echoed the effect of diuretic and excrete dampness. It reflected the guidance of the basic theory of TCM that the four properties, five flavors, channel tropism, and functions and indications were unified. At the same time, the corresponding targets of active components in *W. cocos* acted on different signaling pathways to exert different clinical effects, which reflects the characteristics of multiple components, multiple targets, and multiple pathways of TCM, as well as the basic characteristics of TCM theory, including “the whole concept” and “syndrome differentiation and treatment.” Comprehensive TCM's basic theory and TCM's syndrome level analysis provide a theoretical basis for the determination of the content and main components of different parts of *W*.cocos, which improve the safety and effectiveness of guiding clinical rational drug use.

Therefore, efforts should be paid attention to understand the active components from the perspective of effectiveness, which could be as a reference for screening potential biomarkers based on network pharmacology. To effectively distinguish *Poria and Poriae* cutis samples, HPLC was used to determine the common peak area modeling of different parts. From the perspective of measurability, we usesd chemometrics of PCA, PLS-DA, ResNet, and OPLS-DA to accurately identify different parts of *W. cocos* based on FT-NIR and HPLC. Determination of common components in two parts by standard and determination of their content. However, this approach of HPLC usually comes with a relatively high cost to e?ectively identify different parts of *W. cocos*. In contrast, FT-NIR spectroscopy has the advantages of convenient use, no damage to samples, the lack of cumbersome preprocessing, and fast analysis speed for more information available. These advantages are considered to be a “green” analytical technology. Due to these outstanding features, the method can greatly reduce the loss of samples, avoid reagent contamination, and meet the requirements of sustainable development of resources and energy, which has broad application prospects. The only thing that is missing, this method has several limitations. A good FT-NIR model needs to collect a large number of representative samples in the early stage of model establishment and needs to be continuously updated and expanded to achieve greater practical value. Besides, there are two insufficiencies in this study: (1) although the differences between different parts are greater than between different origins, there is a subsequent need to increase the sample size to identify the different regions of each part to explore the differences in component content between samples of different parts and (2) experimental validation of the efficacy of these three potential biomarkers is still required to explore whether they are associated with diuretic effects, which will provide a scientific explanation and basis for their different efficacy and clinical applications. Integrated with machine learning, fingerprint, and network pharmacology, three components were screened and analyzed as the potential biomarkers for *Poria* and *Poriae* cutis, which may provide a theoretical basis for the effectiveness of clinical medication and the rational utilization of different parts of *W. cocos*.

## Conclusion

In this study, the identification (PCA, PLS-DA, ResNet, and OPLS-DA) models were established combined with machine learning based on FT-NIR and HPLC. From the perspective of measurability, the results showed that the comprehensive chemical quality of samples from different parts was more significantly different than different origins of *W. cocos* with raw and SD FT-NIR. There were some differences in overall chemical information between *Poria* and *Poriae* cutis. The cumulative rate of principal component scores (PC1 and PC2) reaches 98.3%, and the results showed that there is a good classification effect according to different categories. Similarly, the results of the PLS-DA model showed that the classification accuracy of different parts is above 93%. A total of three types of ResNet model results also confirm this conclusion. To further clarify the potential biomarkers of the two parts, the qualitative and quantitative examinations were carried out by HPLC technology and five common peaks were identified. *Poria* and *Poriae* cutis have good similarities, indicating that the method is simple and fast, which can be stably and reliably applied in the quality evaluation process of *W. cocos* in different parts. After VIP > 1 screening, three chemical components (dehydrotrametenolic acid, poricoic acid A, and pachymic acid) were obtained and the different contents have significant differences. Combined with the results of network pharmacology analysis, the above results showed that the 11 main active components of *W. cocos* have played important roles on 6 important targets and five key signaling pathways. It was determined that three chemical components are not only the potential biomarkers that cause differences in different parts but also the active components of *W. cocos* for clinical efficacy. Therefore, through the integrated analysis of fingerprint, machine learning, and network pharmacology, the components of dehydrotrametenolic acid, poricoic acid A, and pachymic acid were screened as the potential biomarkers, which may provide a theoretical basis for rationale developmentg and utilization of non-medicated parts of Yunnan characteristic medicinal plants or fungi, such as *W. cocos*.

## Data Availability Statement

The original contributions presented in the study are included in the article/Supplementary Material, further inquiries can be directed to the corresponding authors.

## Author Contributions

LL conceived, designed the experiments, and wrote the manuscript. ZZ conceived the review, collected literature, and drafted manuscript. YW revised manuscript and provided sample. All authors read and approved the final manuscript for publication.

## Funding

This work was supported by the National Natural Science Foundation of China (Grant Number: 31860584) and the Special Program for the Major Science and Technology Projects in Yunnan Province (Grant Number: 202102AA100010).

## Conflict of Interest

The authors declare that the research was conducted in the absence of any commercial or financial relationships that could be construed as a potential conflict of interest.

## Publisher's Note

All claims expressed in this article are solely those of the authors and do not necessarily represent those of their affiliated organizations, or those of the publisher, the editors and the reviewers. Any product that may be evaluated in this article, or claim that may be made by its manufacturer, is not guaranteed or endorsed by the publisher.
