# Supplementary material for: Practical Qualitative Evaluation and Screening of Potential Biomarkers for Different Parts of Wolfiporia cocos Using Machine Learning and Network Pharmacology
Source: Front Microbiol. 2022 Jul 8;13:931967. doi: 10.3389/fmicb.2022.931967 (PMC9304917; doi:10.3389/fmicb.2022.931967)
Supplement: Supplementary file 1 [file Data_Sheet_1.pdf]

Table S1. Regression equations, linearity ranges, correlation coefficients (R^2), LODs, LOQs and RSD in precision, repeatability, and stability of five reference substances.

Chemical components	Regression equations	r^2	Linearity ranges (μg·mL^{-1})	LODs (μg·mL^{-1})	LOQs (μg·mL^{-1})	Recovery rate (%)	RSD of precision	RSD of repeatability	RSD of stability
Dehydrotumulosic acid	Y = 11319140.93X + 74475.78	0.9983	5.00-999	0.2	0.5	106.4	2.81	5.95	0.58
Poricoic acid A	Y = 12988529.78X + 7769.17	0.9998	2.4-480	0.24	1.5	98.57	2.75	3.51	0.48
Dehydropachymic acid	Y = 7905709.32X + 42996.45	0.9937	10.3-1240	10.33	103.33	100.3	5.68	1.9	0.58
Pachymic acid	Y = 21538210.94X + 107194.87	0.9993	0.49-2450	0.49	2.0	103.7	3.46	1.9	0.71
Dehydrotrametenolic acid	Y = 19016331.45X + 1507413.80 (Poria)	0.9973	0.22-6730	0.22	0.45	96.32	5	1.87	0.55

	Y = 22522136.84X + 42388.59	0.9999	0.22-6730	0.22	0.45	96.32	5	1.87	0.55

Table S2. Confusion matrix for *Poria* and *Poriae* cutis of *W. cocos* based on raw and SD FT-NIR datasets.

Raw dataset (Train set)			
	Poria	*Poriae* cutis	NER (%)
Poria	66	1	98.51%
Poriae cutis	0	67	100%
Total	66	68	99.25%
Raw dataset (test set)			
Poria	33	0	100%
Poriae cutis	1	32	96.97%
Total	34	32	98.48%
SD dataset (Train set)			
	Poria	*Poriae* cutis	NER (%)
Poria	67	0	100%
Poriae cutis	0	67	100%
Total	67	67	100%
SD dataset (test set)			
Poria	33	0	100%
Poriae cutis	2	31	93.94%
Total	35	31	96.97%

Table S3. The results of KEGG enrichment analysis.

Number	KEGG signaling pathway	number of genes	P value
hsa04080	Neuroactive ligand-receptor interaction	13	5.63×10^{-13}
hsa04020	Calcium signaling pathway	8	2.78×10^{-7}
hsa04668	TNF signaling pathway	5	1.79×10^{-4}
hsa05200	Pathways in cancer	7	4.80×10^{-4}
hsa04022	cGMP-PKG signaling pathway	5	7.93×10^{-4}
hsa04270	Vascular smooth muscle contraction	4	3.809×10^{-3}
hsa05219	Bladder cancer	3	5.559×10^{-3}
hsa05140	Leishmaniasis	3	1.6036×10^{-3}
hsa05205	Proteoglycans in cancer	4	1.6639×10^{-3}
hsa05323	Rheumatoid arthritis	3	2.4026×10^{-3}
hsa04915	Estrogen signaling pathway	3	2.9904×10^{-3}
hsa04723	Retrograde endocannabinoid signaling	3	3.1029×10^{-3}
hsa04071	Sphingolipid signaling pathway	3	4.2533×10^{-3}

Figures:

Figure 1. Schematic diagram of residual block.

Figure 2. The residual convolution neural network model used in this study.

Figure 3. Score graph of PCA based on FT-NIR.

Figure 4. Results of the PLS-DA model based on raw and second derivative datasets: (A) optimal number of latent variables; (B) 200 permutation tests; (C) AUC values.

Figure 5. Enrichment analysis of GO.

Figure 6. “Component-target-pathway” Network.

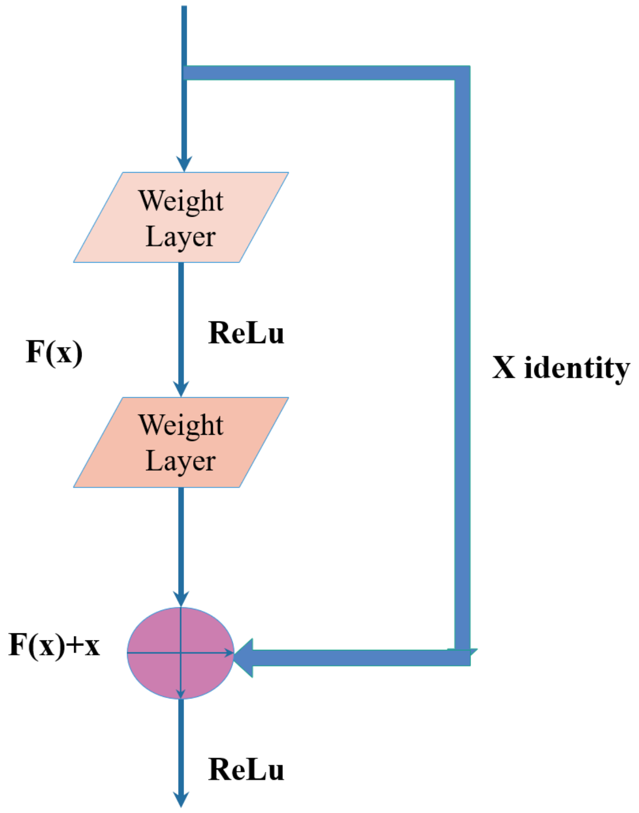

Figure S1. Schematic diagram of residual block.

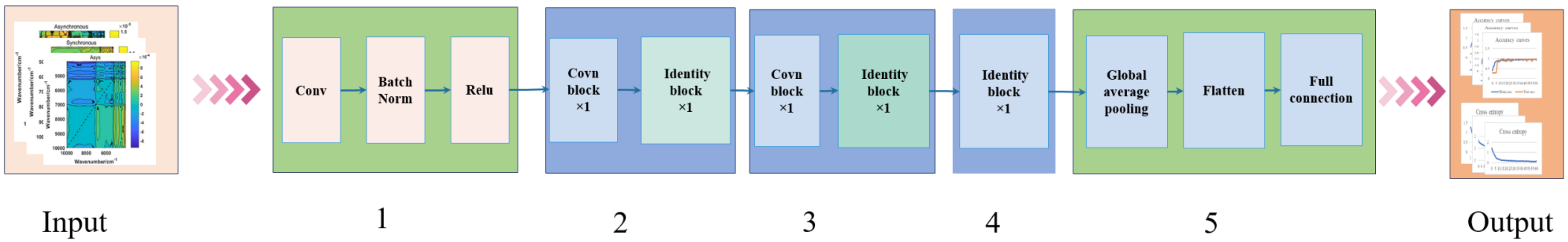

Figure S2. The residual convolution neural network model used in our study.

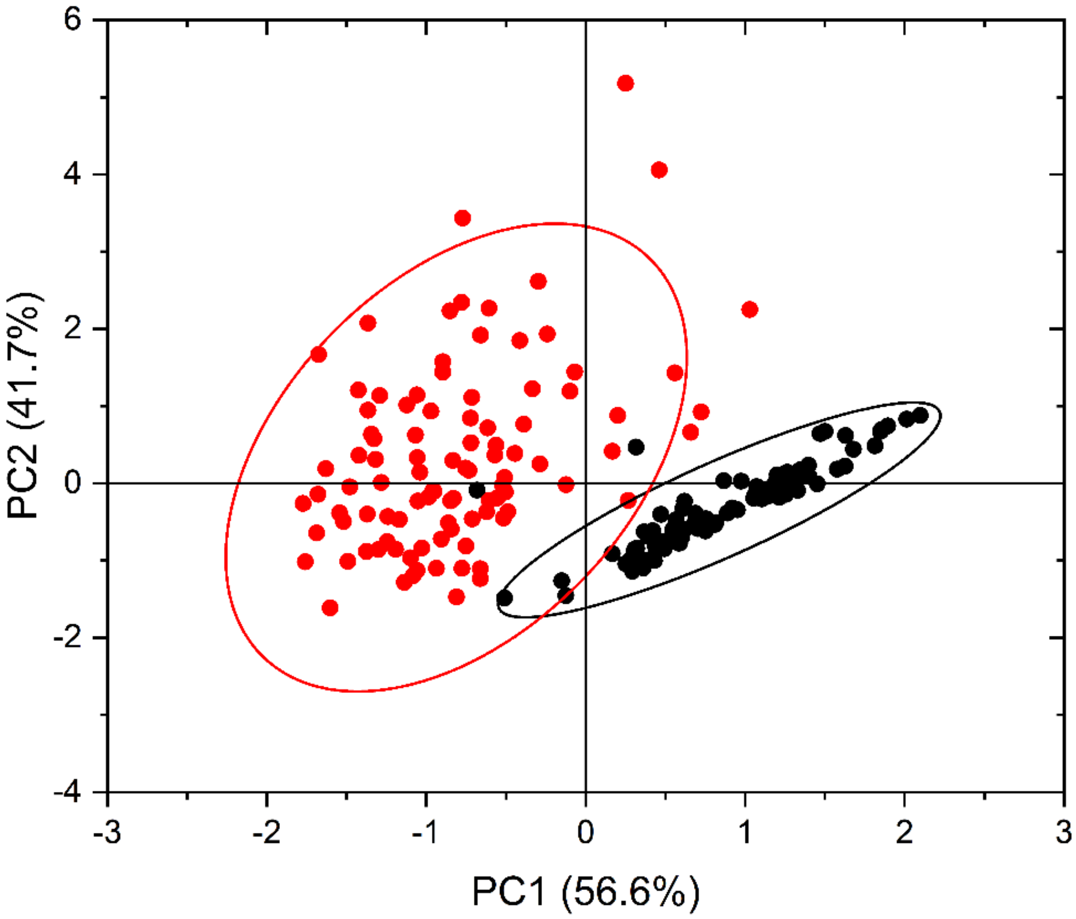

Figure S3. Score graph of PCA based on FT-NIR: The red and black circle represent *Poriae* cutis and *Poria*, respectively.

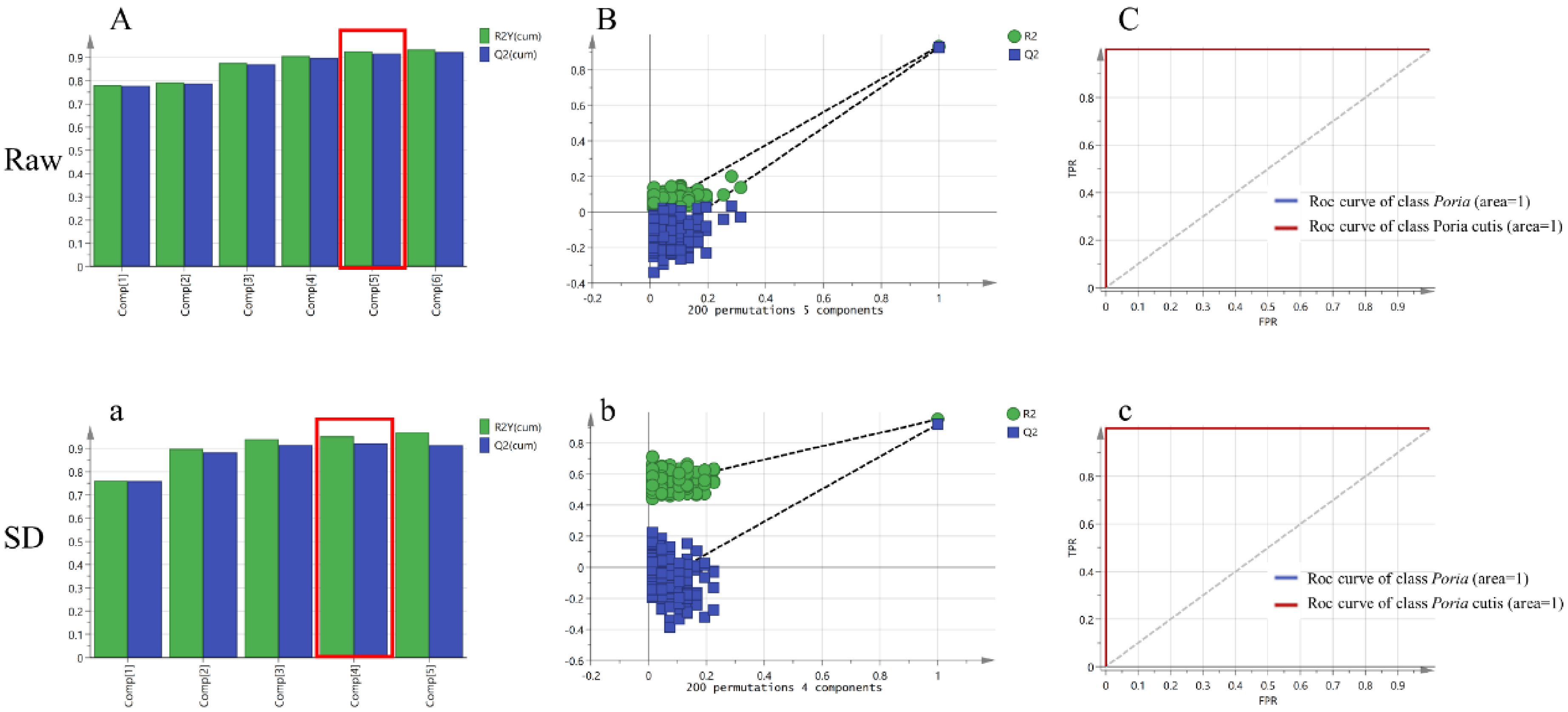

Figure S4. Results of the PLS-DA model based on raw and second derivative datasets: (A) optimal number of latent variables; (B) 200 permutation tests; (C) AUC values.

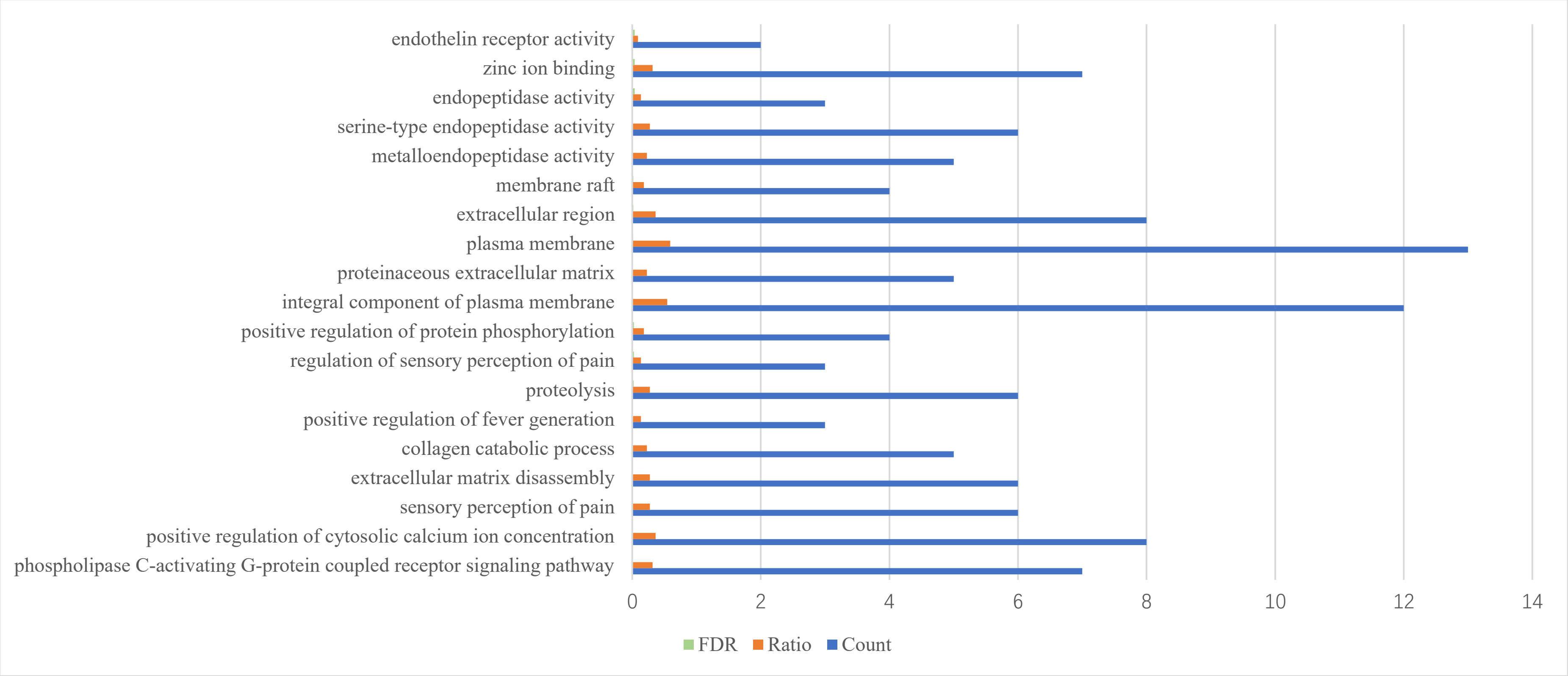

Figure S5. Enrichment analysis of GO.

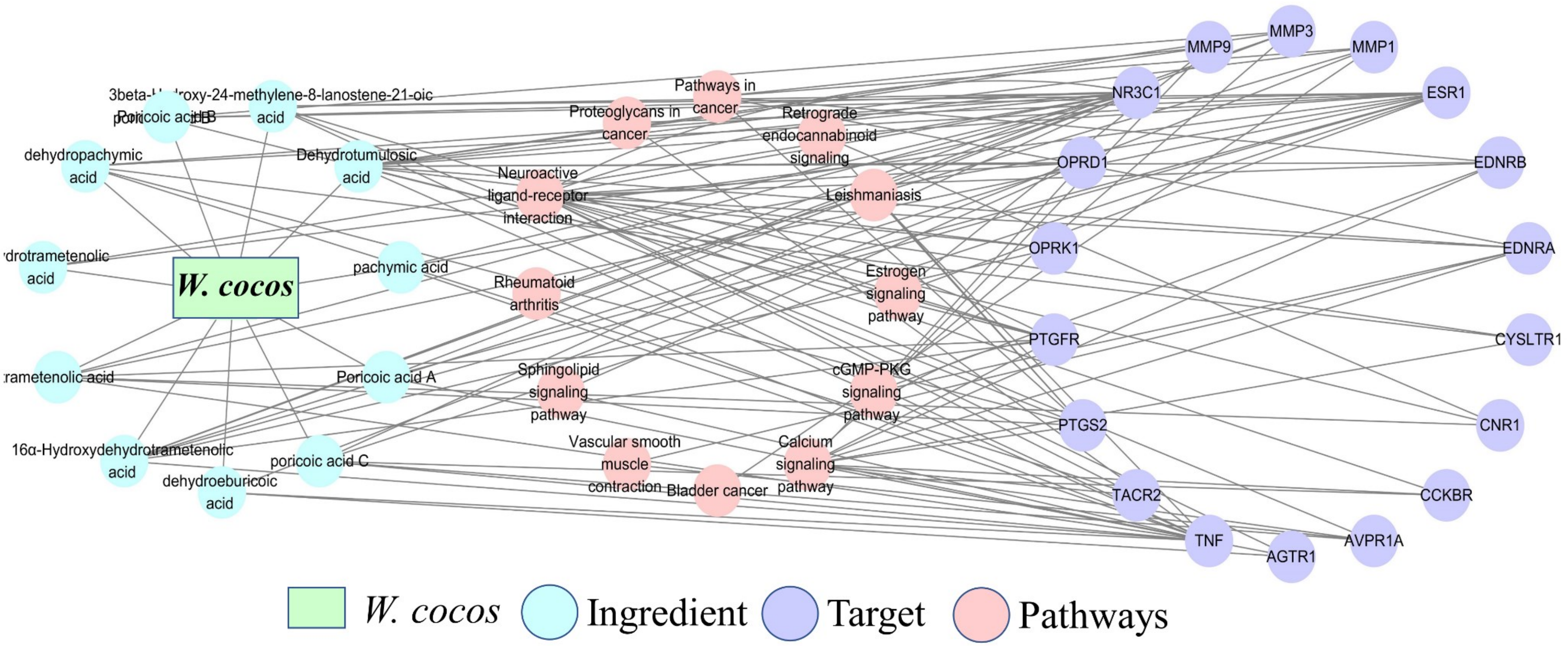

Figure S6. "Component-target-pathway" Network.
